# The Antioxidant N-Acetyl-L-Cysteine Restores the Behavioral Deficits in a Neurodevelopmental Model of Schizophrenia Through a Mechanism That Involves Nitric Oxide

**DOI:** 10.3389/fphar.2022.924955

**Published:** 2022-07-12

**Authors:** Ana Lopes-Rocha, Thiago Ohno Bezerra, Roberta Zanotto, Inda Lages Nascimento, Angela Rodrigues, Cristiane Salum

**Affiliations:** Núcleo Interdisciplinar em Neurociência Aplicada, Centro de Matemática, Computação e Cognição, Universidade Federal do ABC, São Bernardo do Campo, Brazil

**Keywords:** schizophrenia, N-acetyl-L-cysteine, MAM model, neuroinflammation, oxidative stress, nitric oxide, GFAP, Iba

## Abstract

The disruption of neurodevelopment is a hypothesis for the emergence of schizophrenia. Some evidence supports the hypothesis that a redox imbalance could account for the developmental impairments associated with schizophrenia. Additionally, there is a deficit in glutathione (GSH), a main antioxidant, in this disorder. The injection of metilazoximetanol acetate (MAM) on the 17th day of gestation in Wistar rats recapitulates the neurodevelopmental and oxidative stress hypothesis of schizophrenia. The offspring of rats exposed to MAM treatment present in early adulthood behavioral and neurochemical deficits consistent with those seen in schizophrenia. The present study investigated if the acute and chronic (250 mg/kg) treatment during adulthood with N-acetyl-L-cysteine (NAC), a GSH precursor, can revert the behavioral deficits [hyperlocomotion, prepulse inhibition (PPI), and social interaction (SI)] in MAM rats and if the NAC-chronic-effects could be canceled by L-arginine (250 mg/kg, i.p, for 5 days), nitric oxide precursor. Analyses of markers involved in the inflammatory response, such as astrocytes (glial fibrillary acid protein, GFAP) and microglia (binding adapter molecule 1, Iba1), and parvalbumin (PV) positive GABAergic, were conducted in the prefrontal cortex [PFC, medial orbital cortex (MO) and prelimbic cortex (PrL)] and dorsal and ventral hippocampus [CA1, CA2, CA3, and dentate gyrus (DG)] in rats under chronic treatment with NAC. MAM rats showed decreased time of SI and increased locomotion, and both acute and chronic NAC treatments were able to recover these behavioral deficits. L-arginine blocked NAC behavioral effects. MAM rats presented increases in GFAP density at PFC and Iba1 at PFC and CA1. NAC increased the density of Iba1 cells at PFC and of PV cells at MO and CA1 of the ventral hippocampus. The results indicate that NAC recovered the behavioral deficits observed in MAM rats through a mechanism involving nitric oxide. Our data suggest an ongoing inflammatory process in MAM rats and support a potential antipsychotic effect of NAC.

## 1 Introduction

Schizophrenia affects around 0.3% of the world population (Charlson et al., 2018) and is characterized by hallucination, delusion, social anhedonia, and cognitive deficits (Kahn et al., 2015). Some studies point to a developmental origin of schizophrenia, showing that problems during the gestation involving neuroinflammation and oxidative stress can increase the risk of developing this disorder (Stilo and Murray, 2019; Ermakov et al., 2021). Patients with schizophrenia show a reduction in blood and anterior cingulate cortex concentration of glutathione (GSH), one of the main antioxidants of the brain (Tsugawa et al., 2019). Accordingly, treatment with N-acetyl-L-cysteine (NAC), a precursor of GSH, has shown improvement in schizophrenia symptoms (Cho et al., 2019).

The increased oxidative stress and brain inflammatory response can cause functional alteration in brain circuits in schizophrenia (Dwir et al., 2020). The subclass of GABAergic interneurons that express parvalbumin (PV) is altered in schizophrenia, with post-mortem studies showing a reduction in tissue in the prefrontal cortex (PFC) and hippocampus (Beasley and Reynolds, 1997; Zhang and Reynolds, 2002) and hypermethylation of PV promoter gene in the hippocampus (Fachim et al., 2018), when compared to control subjects. The reduction of PV positive interneurons was associated with the increased activity in the ventral hippocampus detected in patients with this disorder (McHugo et al., 2019), possibly leading to the symptoms of schizophrenia (Grace and Gomes, 2019). Some studies with the animal models of schizophrenia showed that the increased oxidative stress could be a causing factor to the reductions in the PV positive interneurons. Indeed, the animal model with knockout for the glutamate–cysteine ligase regulatory subunit gene (GCLM), an enzyme involved in GSH synthesis, showed behavioral impairments and reduction of PV interneurons in the PFC and hippocampus (Kulak et al., 2012; Dwir et al., 2021).

It is important to note that glial cells, such as astrocytes and microglia, can modulate and mediate the oxidative stress and inflammatory responses in the brain (Giovannoni and Quintana, 2020). A study with post-mortem tissue of patients with schizophrenia found an association between increased GFAP (glial fibrillary acidic protein, an astrocyte marker) protein and mRNA with neuroinflammation (Catts et al., 2014). Some animal models of schizophrenia also presented increased GFAP positive astrocytes in the frontal cortex and hippocampus (Kim et al., 2018). Since astrocytes modulate the inflammatory response in the brain (Giovannoni and Quintana, 2020) and the microglia mediates that process, these cells could be involved in the oxidative stress and neuroinflammation in schizophrenia. A study of patients with schizophrenia found an increased density of cells expressing the ionized calcium-binding adapter molecule 1, a microglia marker (Iba1), and positive cells in the post-mortem brain in the frontal, cingulate, and temporal cortex when compared to control subjects (Gober et al., 2022).

The methylazoxymethanol (MAM) model of schizophrenia in rats recapitulates several hallmarks of schizophrenia. Pregnant rats received an injection of MAM at the 17° day of gestation, temporarily interrupting the neurodevelopment of the offspring (Grace and Gomes, 2019). Among the various behavioral deficits observed in rats whose mothers received MAM, hyperlocomotion induced by a psychotomimetic agent, deficits in social interaction (SI) and in prepulse inhibition (PPI) tests are observed in their adult life (Moore et al., 2006; Perez et al., 2019a). Those rats also showed a reduction in the PV positive interneurons density in the ventral hippocampus and PFC (Penschuck et al., 2006; Du and Grace, 2016; Perez et al., 2019b), increased ventral tegmental area activity, oxidative stress, and neuroinflammatory markers (Zhu et al., 2021). Interestingly, the treatment with NAC during the prepubertal stage of development prevented the increased activity of the ventral tegmental area in MAM rats (Zhu et al., 2021).

However, it is not clear if the treatment with NAC during adulthood could also recover the behavioral and cellular alterations detected in the MAM model of schizophrenia. Hence, the objective of the present study was to evaluate the acute and chronic treatments with NAC over the hyperlocomotion, SI, and PPI tests of adult MAM rats. As we found a recovery of the MAM impaired behaviors with NAC treatments, we then investigated if the NAC mechanism of action involved nitric oxide. Additionally, we evaluated the effects of MAM and/or NAC chronic treatment on the density of PV positive interneurons, GFAP positive astrocytes, and Iba1 positive microglia in the hippocampus and PFC after chronic NAC treatment.

## 2 Materials and Methods

### 2.1 Animals

Wistar rats (University of São Paulo, Institute of Biomedical Science, Brazil), with approximately 85 days and weighing about 300 g, were subjected to the mating procedure: female (*N* = 16) rats in the proestrus or estrus phase were maintained with one sexually experienced male (*N* = 8). Pregnancy was verified by vaginal smear and sperm detection. These females were housed in at most two per cage. All the rats were housed in cages of polypropylene walls (40.0 cm × 33.0 cm × 18.0 cm), with 3.0 cm of sawdust and controlled condition of temperature (23.0 ± 1.0°C) and light (12/12 h light/dark cycle, beginning at 7 a.m.). The rats had food and water ad libitum. All the experiments were performed in the laboratories of the Interdisciplinary Nucleus of Applied Neuroscience at the Federal University of ABC (UFABC), São Bernardo do Campo, Brazil and the procedures had the ethical approval of the Ethics Committee of UFABC (CEUA/UFABC, protocol 007/2014 and 8946130619/2019). All behavioral tests were conducted in rats at postnatal days (PN) 90 or 91. After completion of behavioral experiments, each rat was euthanized with an injection of urethane (Sigma, 3 mg/kg, i.p.).

### 2.2 Drugs

Antimitotic acetate of metilazoximetanol (MAM, Midwest Research Institute, Kansas City, United States ) was dissolved in saline (0.9%) and administered i.p. at a dose of 25 mg/kg and 1 ml/kg (Lodge et al., 2009). NAC (Sigma-Aldrich, United States ), an antioxidant precursor of GSH, was dissolved in saline and NaOH was used to set pH at 6.0 (Fukami et al., 2004; Khan et al., 2004). NAC was administered i.p. at the doses of 150, 250, and 500 mg/kg at the volume of 1 ml/kg (Dean et al., 2011; Möller et al., 2013). L-arginine (Sigma-Aldrich, United States ), a nitric oxide precursor, was dissolved in saline and administered i.p. at a dose of 250 mg/kg at 1 ml/kg (Johansson et al., 1997).

### 2.3 Experimental Design

#### 2.3.1 MAM Treatment

At the 17° gestational day (GD17), pregnant rats received either an injection of MAM (25 mg/kg, i.p) or saline above the midline, to avoid reaching gestational sacs, and they were placed individually in separate cages. After birth (between GD21 to GD23) and weaning period (PN21), male offsprings were separated from their mothers and housed, with four rats per cage. In experiment 1, male offsprings received one single injection of either NAC or saline at PN90, and in experiment 2, male offsprings received 15 days of treatment with NAC or saline, starting at PN75. In experiment 2, male offsprings also received a sub-chronic treatment of L-arginine or saline for 5 days, starting at PN85.

#### 2.3.2 Experiment 1—Acute Effect of NAC in Male Rats of the MAM Model

At PN90, adult MAM and saline rats (*N* = 36) were subdivided into four subgroups each, which then received either an injection of saline or NAC (150, 250, or 500 mg/kg and volume of 1 ml/kg, i.p.), one hour before the PPI test. Following that test, the rats were subjected to SI and then to hyperlocomotion tests.

#### 2.3.3 Experiment 2—Chronic Effect of NAC (250 mg/kg) and/or L-Arginine in the Rats of MAM Model

From PN75 to PN90, adult MAM and saline rats (*n* = 44) were divided into two subgroups each, which then received a daily injection of NAC (250 mg/kg, at 1 ml/kg, i.p.) or saline for 15 days. From the PN85th day of life, these rats were subdivided again into two groups receiving daily injections of L-arginine (250 mg/kg, at 1 ml/kg, i.p.) or saline for 5 days. One day after the last injection of both treatments (PN91), they were subjected to SI and hyperlocomotion tests.

### 2.4 Behavioral Tests

#### 2.4.1 Prepulse Inhibition of Startle

The tests were conducted in a sound-attenuating startle box chamber (Insight Equipamentos, Brazil) of plywood (64.0 cm × 60.0 cm × 40.0 cm) ventilated by a fan at the top of the chamber. In the center, there was a wire-mesh stabilimeter cage (16.5 cm × 5.1 cm × 7.6 cm) suspended within a PVC frame (25.0 cm × 9.0 cm × 9.0 cm) and attached to the response platform by four thumbscrews. The floor of the cage consisted of six stainless steel bars with a 3.0 mm diameter spaced 1.5 mm apart. The startle reaction of the rats generated a pressure on the response platform and analog signals were amplified, digitalized, and analyzed by a software of the startle measure system. Two loudspeakers located 10 cm above the floor on each side of the acoustic chamber presented all sound stimuli. Calibration procedures were conducted daily before the experiments to ensure equal sensitivity of the response platform over the test period.

At least 2 days prior to the experiments, all rats were subjected to the PPI test described in the following section for the matching procedure. This process ensures a homogeneous animal distribution by baseline PPI among treatment groups and provides greater data reliability.

Each rat was placed in a startle chamber with background noise of 57 dB. The PPI session began with the presentation of 10 pulses (white noise, 120 dB, 40 ms) to determine the baseline startle and produce startle habituation. In sequence, 72 trials were divided into pseudorandom presentations of eight different types of stimuli: pulse (P), prepulse (PP, 20 ms pure tone at the frequency of 3,000 Hz and intensities of 69, 73, and 81 dB), pulse preceded by prepulse (PP + P, with an interstimulus interval of 100 ms), and no stimuli (background noise, 57 dB). The average intertrial interval was 20 s during the habituation block and 30 s during the rest of the session.

#### 2.4.2 Social Interaction in the Open Field

The open field consisted of a cylindrical arena of transparent acrylic 50.0 cm (height) × 60.0 cm (diameter) with a wooden base (100.0 cm × 80.0 cm) painted in matte black. After the PPI test, each rat was placed in the open field for exploration and habituation for 5 min. Following that, each rat was placed in the open field containing an unfamiliar male rat under the same treatment, for the SI test. The rats were positioned at opposite sides of the open field. All behaviors performed for 10 min were recorded by a camera positioned above the equipment and connected to a computer with an EthoVision System (Noldus, Netherlands). The behaviors analyzed were divided into active interaction (sniffing, following, anal/genital inspection, and mounting) and passive interaction (when the rat was allowed to get closer), and the total time of interaction was evaluated.

#### 2.4.3 Hyperlocomotion Induced by Psychostimulant

After the SI test, each rat received a subcutaneous injection of saline or the NMDA receptor antagonist MK-801 (0.05 mg/kg, Sigma-Aldrich, United States ), 40 min before the test. Each rat was placed individually in the open field to explore it for 30 min. During the entire test, a low light was used to illuminate the room. The trial was recorded, and the total distance traveled (cm) was analyzed by EthoVision (Noldus, Netherlands). In experiment 2, all animals received MK-801 injection before hyperlocomotion test.

### 2.5 Immunohistochemistry

After the behavioral tests, all the rats were euthanized with urethane (1,500 mg/kg, i.p.) and perfused transcardially with saline (200 ml) and paraformaldehyde (200 ml, 4% w/v). The rats were decapitated and their brains were removed, postfixed for 2 h (4% w/v), and cryoprotected in sucrose for 42 h (30% w/v in 0.1 M phosphate buffer). The brain slices of the rats under chronic treatment with NAC were made in a cryostat (Leica, Germany), comprising coronal sections with 40 µm taken from the hippocampus (bregma: −4.56 to −6.12 mm) and PFC (bregma: 3.72–5.16 mm). The sections were first rinsed (3 × 5 min) in phosphate buffer (PBS) + 0.15% TritonX-100 (pH 7.4, washing buffer) and incubated in sodium citrate buffer (pH 6.0, 1 × 5 min). Next, they were incubated a second time with a sodium citrate buffer at heating temperature (60°C, 1 × 30 min). After that, the slices were pre-incubated with hydrogen peroxide 1% in 0.1 PBS (1 × 30 min) to block endogenous peroxidase. The slices were then rinsed again in the washing buffer (3 × 5 min) and incubated in 2% bovine serum albumin + 5% normal goat serum for GFAP and Iba1 or 5% normal horse serum for PV prepared in the washing buffer (1 × 60 min). The slices were incubated overnight with primary polyclonal antibodies for GFAP (rabbit, 1:1,000, Z0334, Dako, Denmark), PV (mice, 1:1,000, P227, Sigma-RBI, EUA), or Iba1 (rabbit, 1:500, Thermo Fisher Scientific, PA5-27436, United States ). Subsequently, the slices were rinsed again in the washing buffer (3 × 5 min) and incubated in goat anti-rabbit (1:400, ThermoFisher Scientific, EUA, for GFAP and IBA1) or horse anti-mouse (1:400, Vector Laboratories, for PV) biotinylated secondary antibodies (1 × 60 min). Once removed, the slices were successively washed with a washing buffer (3 × 5 min) and then with the biotin–avidin-peroxidase complex (1:300, Vectastain ABC Kit, Vector Laboratories, EUA; 1 × 120 min). Immunoreactions were revealed using 3,3’-diaminobenzidine + hydrogen peroxide 0.02% (DAB, Sigma Aldrich, EUA) in saline tris (hydroxymethyl) aminomethane 0.1 M (TBS). All reactions were conducted at 21°C under agitation. The slices were observed under an optical microscope (Leica, D5500M, Germany) with the amplification of 5×. The structures evaluated were: CA1, CA2, CA3, dentate gyrus (DG), and ventral subiculum (Sub) from the dorsal or ventral hippocampus and medial orbital cortex (MO) and prelimbic cortex (PrL) from PFC (Paxinos and Watson, 2006). The total density in each region was calculated by taking the sum of all the positive cells in each region divided by the entire area. The density of all positive immunoreactive cells for PV, GFAP, and Iba1 in each area was obtained using ImageJ.

### 2.6 Statistical Analysis

In experiment 1, statistical analysis was developed with factors: treatment 1: MAM × saline, treatment 2: NAC1 (150 mg/kg), NAC2 (250 mg/kg), and NAC3 (500 mg/kg) × saline, and treatment 3: MK-801 × saline. In experiment 2, factors are: treatment 1: MAM × saline, treatment 2: NAC × saline, and treatment 3: L-arginine × saline.

The total distance traveled (cm) for each rat in the hyperlocomotion test was analyzed for both experiments by a three-way ANOVA considering treatment 1, treatment 2, and treatment 3 as between subjects’ factors. In the SI test, total (active plus passive) interaction time was analyzed by a two-way ANOVA in experiment 1, with treatment 1 and treatment 2 as between subjects’ factors, and three-way ANOVA in experiment 2, with treatment 1, treatment 2, and treatment 3 as between subjects’ factors.

On the PPI test, mean ASR to pulse-alone (P) and prepulse-pulse (PP + P) trials were calculated for each subject. The %PPI (percentage of ASR to PP + P related to P) was calculated by.

$$\%PPI=100-\left[100\times \left(\frac{PP+P}{P}\right)\right].$$

The %PPI was analyzed by a repeated measure ANOVA with stimulus intensity (69, 73, and 81 dB) as within subjects’ factor, and treatment 1 and treatment 2 as between subjects’ factors. Similarly, an analysis was made of the ASR with the stimulus as a within subjects’ factor (P, PP69 + P, PP73 + P, and PP81 + P).

For immunohistochemistry results, the statistical analysis was carried out with a two-way ANOVA and treatment 1 and treatment 2 as between subjects’ factors.

Statistical analyses were performed using the statistical package SPSS (version 20, IBM), and considered statistically significant differences at *p* < 0.05. When necessary, there was *post hoc* analysis with multiple comparisons using the Duncan test to explore interactions that might reveal significant differences between specific groups.

## 3 Results

### 3.1 Experiment 1—NAC Acute Effect in Male Rats of MAM Model

#### 3.1.1 Hyperlocomotion Induced by the NMDA Antagonist

**FIGURE 1:**
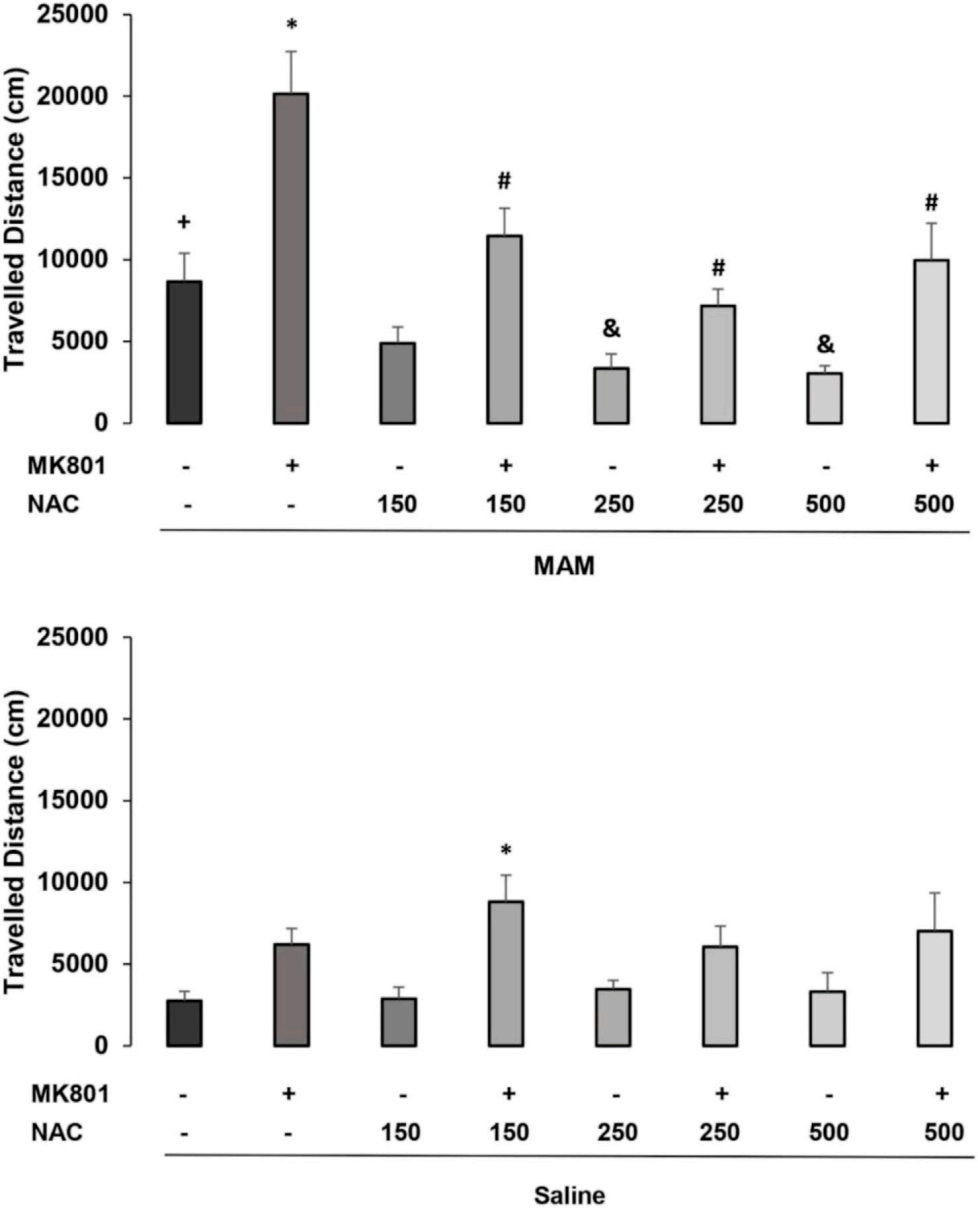
Effect of acute NAC treatment (150 , 250, and 500 mg/kg) in the MAM model on the distance (cm) traveled at the open field (mean ± SE). Adult male offspring of MAM- or saline (Sal)-treated rats received at adulthood a single NAC (or saline) injection (1 h before) and an injection of MK801 (0.05 mg/kg) or saline, 40 min before testing. * indicates significant difference from the Sal/Sal/MK group. # indicates significant difference from group MAM/Sal/MK. + indicates significant difference from the group Sal/Sal/Sal and & indicates significant difference compared to the MAM/Sal/Sal group (Duncan, *p* < 0.05).

A three-way ANOVA revealed significant main effects for treatment 1 (MAM or saline) [F (1, 98) = 22.179; *p* < 0.01], treatment 2 (150 mg NAC, NAC 250 mg, 500 mg or NAC saline) [F (3, 98) = 6.934; *p* < 0.01], and treatment 3 (MK or saline) [F (1, 98) = 55.415; *p* < 0.01], and interactions between treatment 1 × treatment 2 [F (3, 98) = 8.774; *p* < 0.01] and treatment 1 × treatment 3 [F (1, 98) = 4.875; *p* < 0.05]. The *post hoc* Duncan analysis showed that the group treated with MAM/Sal/MK differed significantly from the MAM/Sal/Sal group (*p* < 0.05), while the group Sal/Sal/MK did not differ from the Sal/Sal/Sal group (Figure 1). MAM rats presented hyperresponsiveness to the sub-dose of the MK-801, which was significantly reduced by the treatment with NAC at all doses. This was not observed in the saline animals. MAM rats also showed significantly increased locomotion, compared to the control group, and the doses of 250 and 500 mg of NAC were able to reduce this effect (*p* < 0.05). Although MK801 did not cause a significant increase in locomotion of the control group, when it was administered with NAC at the dose of 150 mg, there was an unexpected significant increase in locomotion, compared to control (*p* < 0.05, Duncan).

#### 3.1.2 Social Interaction

**FIGURE 2:**
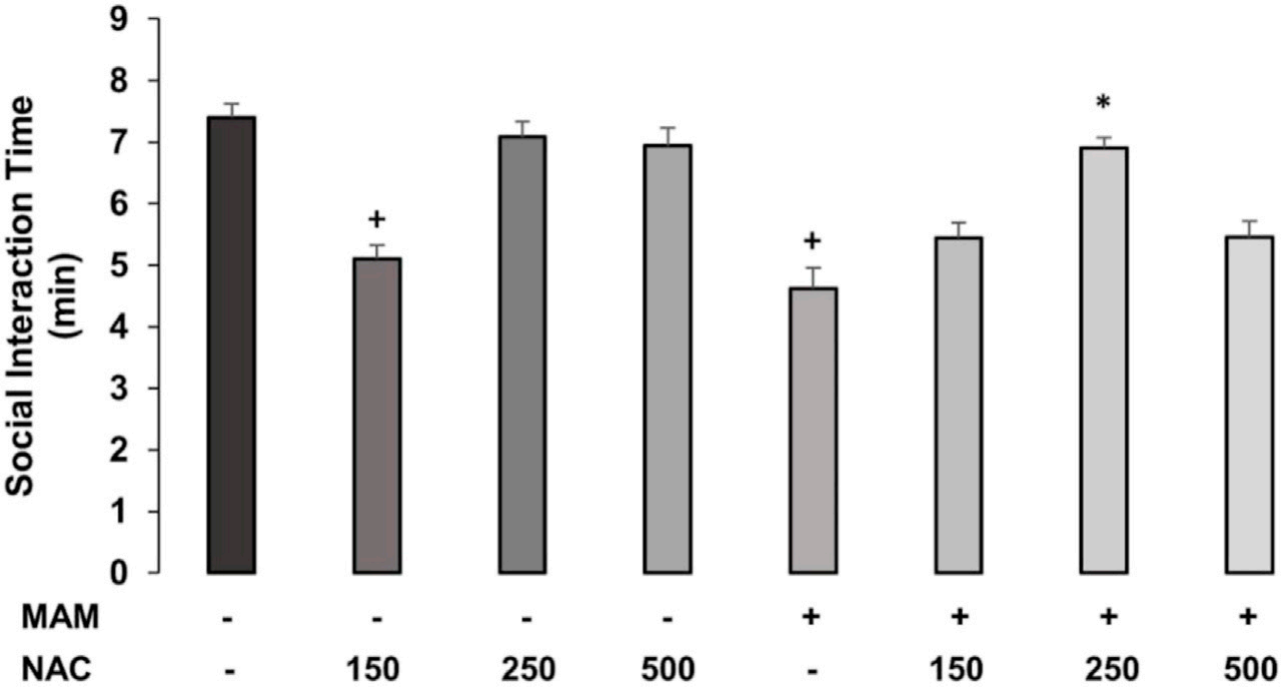
Time (min) spent (mean ± SE) on active and passive social interaction behaviors in MAM rats treated with NAC (150 mg, 250 mg, and 500 mg/kg). Adult male offspring of MAM- or Sal-treated rats received a single NAC (or saline) injection at one of the doses and were placed in the open field with another unfamiliar rat, under the same treatment, for 10 min. + indicates significant difference compared to the Sal/Sal group. * indicates a significant difference related to the MAM-saline group (Duncan, *p* < 0.05).

The two-way ANOVA for time spent in SI behaviors showed the main effects of treatment 1 (MAM × Sal) [F (1, 62) = 3.775; *p* = 0.05] and treatment 2 (NAC × Sal) [F (4, 62) = 17.362; *p* < 0.01], and interaction between treatment 1 × treatment 2 [F (2, 62) = 5.199, *p* < 0.05] (Figure 2). The *post hoc* analysis of Duncan revealed that MAM rats showed significantly reduced social interaction time, compared to saline rats, and NAC treatment at the dose of 250 mg/kg was able to raise this behavior in MAM rats (*p* < 0.05). The dose of 150 mg/kg of NAC did not affect the behavior of MAM rats, but decreased the time of SI behavior in saline rats, while the other two doses did not change these behaviors in the control group.

#### 3.1.3 Prepulse Inhibition

**FIGURE 3:**
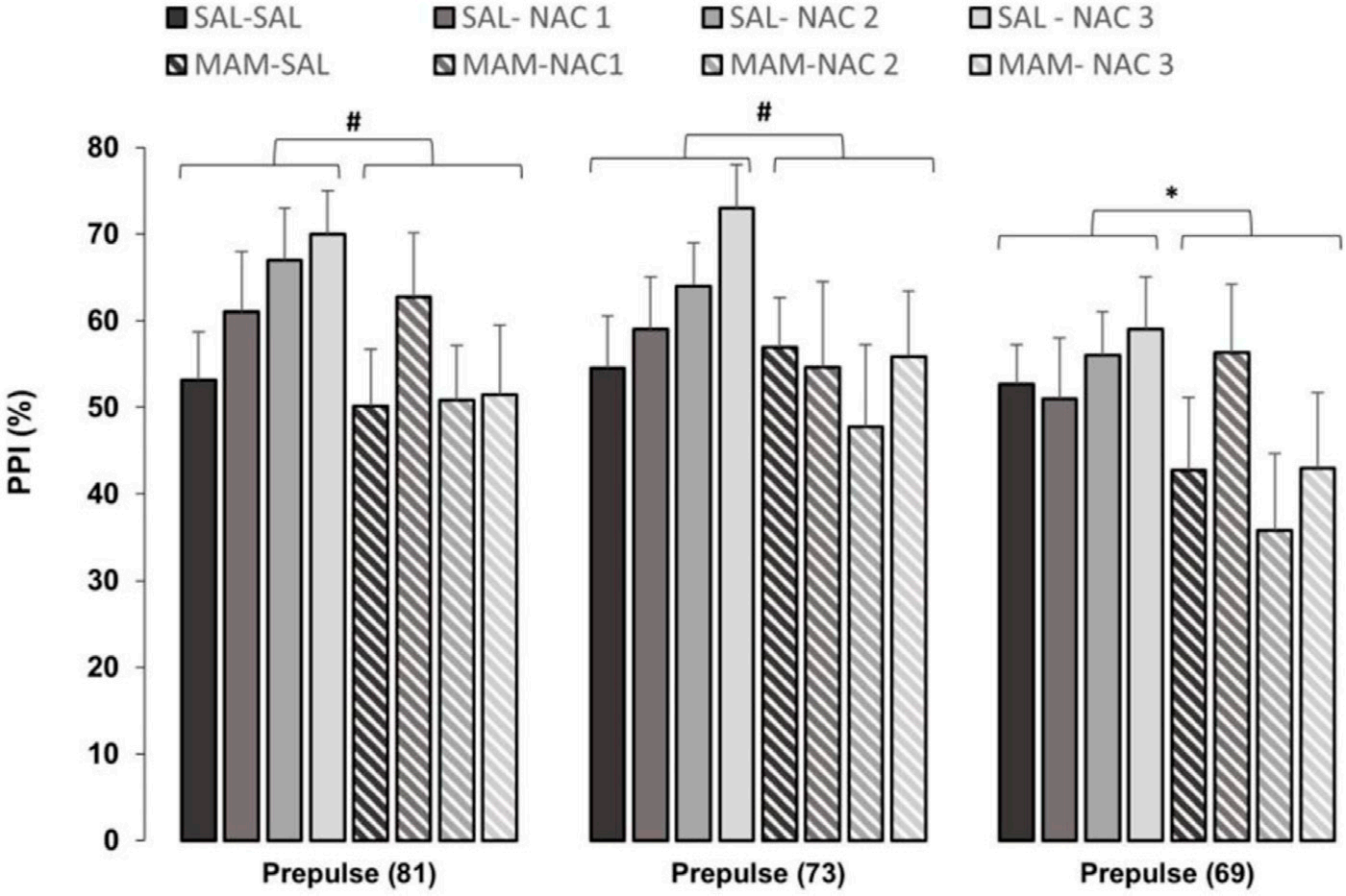
Effect of NAC1, NAC2, or NAC3 (150, 250, and 500 mg/kg, respectively) on the MAM model at %PPI (mean ± SE). Adult male offspring of MAM- or Sal-treated rats received a single injection of NAC1, NAC2, or NAC3 and were placed at the chamber for a PPI session, consisting on presentation of 10 pulses (P, 120 dB), then 72 trials divided into: P, prepulse (PP of 69, 73, and 81 dB), P preceded by PP (interstimulus interval of 100 ms), and no stimuli (background noise, 57 dB). * Indicates significant difference between MAM and Sal groups (Duncan, *p* < 0.05). # Indicates marginal differences between MAM and Sal groups (Duncan, prepulse (81): *p* = 0.063; prepulse (73): *p* = 0.071).

A three-way repeated measures ANOVA, with treatment 1 and treatment 2 as between subjects factors and intensity (of prepulse, 69, 73, and 81 dB) as within subjects for %PPI showed the significant main effects of treatment 1 (MAM × Sal) [F (1,121) = 5.136; *p* < 0.05] and of intensity [F (2, 242) = 10.776; *p* < 0.001]. The Duncan *post hoc* test showed a statistically significant effect of treatment 1 on the intensity of 69 dB (*p* < 0.05) and marginal effects with the intensities of 73 dB (*p* = 0.071) and 81 dB (*p* = 0.063) (Figure 3).

#### 3.1.4 Acoustic Startle Response

**FIGURE 4:**
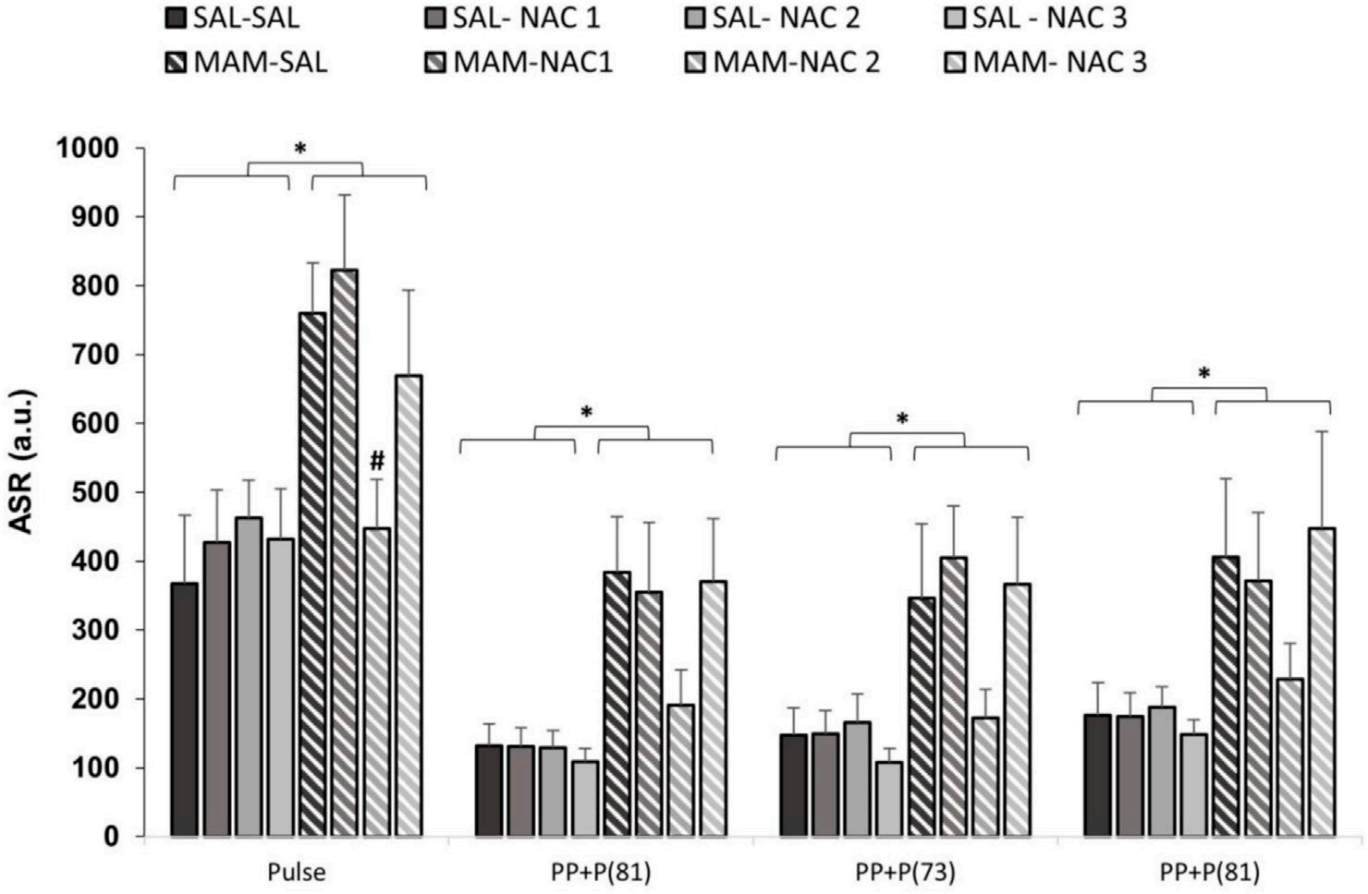
Effect of NAC1, NAC2, or NAC3 (150, 250, and 500 mg/kg, respectively) on the MAM model at the acoustic startle response (ASR) (mean ± SE) to the stimulus. Adult male offspring of MAM- or saline- (Sal) treated rats received a single NAC1, NAC2, or NAC3 injection and were placed at the chamber for a PPI session, consisting on presentation of 10 pulses (P, 120 dB), then 72 trials divided into: P, prepulse (PP of 69, 73 and 81 dB), P preceded by PP (interstimulus interval of 100 ms) and no stimuli (background noise, 57 dB). * indicates significant difference when compared to SAL groups. # indicates significant difference when compared to the MAM/Sal group (Duncan, *p* < 0.05).

The three-way repeated measures ANOVA, with treatment 1 and treatment 2 as between subjects factor and intensity (pulse of 120 dB and prepulses of 69, 73, and 81 dB) as within subjects, for ASR to stimuli revealed the main effects of treatment 1 (MAM × saline) [F (1, 121) = 16.803; *p* < 0.001] and stimulus [F (3, 363) = 129.590; *p* < 0.001]. The Duncan *post hoc* test showed that MAM rats had an ASR significantly higher than that of the saline group to all stimuli intensity (*p* < 0.05), except for PP73 (*p* = 0.081) (Figure 4). The NAC (250 mg/kg) reduced the responses to the pulse (*p* < 0.05) in the MAM/NAC2 group compared to the MAM/SAL group. The analysis also revealed that NAC did not cause any significant change in the ASR in saline groups.

### 3.2 Experiment 2—Effect of Chronic Treatment With NAC (250 mg/kg) and/or L-Arginine in MAM Rats

#### 3.2.1 Social Interaction

**FIGURE 5:**
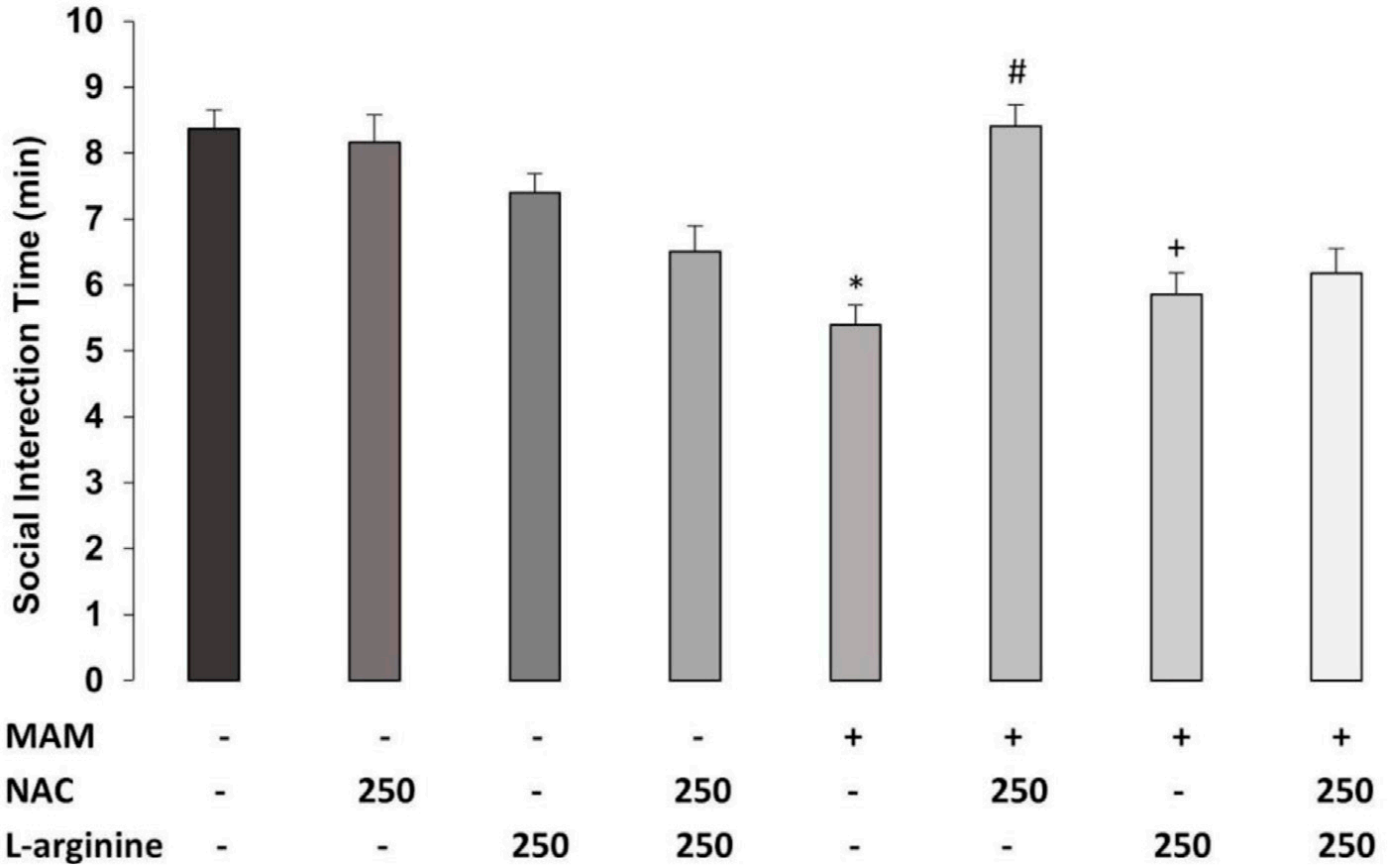
Effect of chronic treatment (15 days) with NAC (250 mg/kg) and/or L-arginine (5 days) (250 mg/kg) on the time (min) of social interaction (SI) behaviors (mean ± SE) on the MAM model. Adult male offspring of MAM- or saline (Sal)-treated rats, chronically treated with NAC or Sal and/or sub-chronically with L-arginine or Sal, were placed in an open field with an unfamiliar male rat under the same treatment, for 10 min * indicates significant difference compared to the Sal/Sal/Sal group. # indicates significant difference from the MAM/Sal/Sal group. + indicates significant difference from MAM/NAC/Sal (Duncan, *p* < 0.05).

The three-way ANOVA for time of SI revealed the main effects of treatment 1 (MAM × Sal) [F (1, 36) = 22.559; *p* < 0.01], treatment 2 (NAC × Sal) [F (1, 36) = 5,271; *p* < 0.05], and treatment 3 (L-arginine × Sal) [F (1, 36) = 20.409; *p* < 0.01]. There were also statistically significant interactions between treatment 1 × treatment 2 [F (1, 36) = 20.918; *p* < 0.01] and treatment 2 × treatment 3 [F (1, 36) = 12.241; *p* < 0.01]. The Duncan *post hoc* test revealed that MAM treatment caused a reduction on SI time, compared to Sal rats (*p* < 0.05) (Figure 5). Chronic treatment with NAC (250 mg/kg) increased the time of SI in MAM rats (*p* < 0.05). However, the sub-chronic treatment with L-arginine reduced the SI time on MAM rats treated with NAC (*p* < 0.05), showing that the NO precursor was able to block the effect of NAC in MAM rats.

#### 3.2.2 Hyperlocomotion Induced by the NMDA Antagonist

**FIGURE 6:**
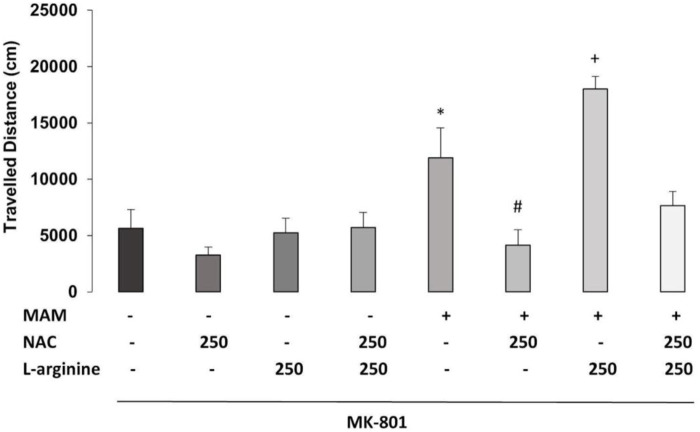
Effect of chronic treatment (15 days) with NAC (250 mg/kg) and/or L-arginine (5 days) (250 mg/kg) on the traveled distance (cm) at the arena (30 min) (mean ± SE) on the MAM model. Adult male offspring of MAM- or saline (Sal)-treated rats, chronically treated with NAC or Sal and/or sub-chronically with L-arginine or Sal, received an MK801 (0.05 mg/kg) injection 40 min before they were placed on the center of an open field, for 30 min exploration. * indicates significant difference compared to the group Sal/Sal/Sal. # indicates significant difference compared to the MAM/Sal/Sal group. + indicates a significant difference compared to all other groups (Duncan, *p* < 0.05).

The three-way ANOVA found statistically significant main effects of treatment 1 (MAM × Sal) [F (1, 36) = 26.913; *p* < 0.01], treatment 2 (NAC × Sal) [F (1, 36) = 22.481; *p* < 0.01], and treatment 3 (L-arginine × Sal) [F (1, 36) = 7.682, *p* < 0.05]. There was a statistically significant interaction between treatment 1 × treatment 2 [F (1, 36) = 14.868; *p* < 0.01] (Figure 6). Duncan’s *post hoc* analysis revealed that MAM rats showed a higher locomotor activity, compared to saline rats, all under the treatment with a sub-dose of MK-801 (*p* < 0.05). This hyperresponse to MK-801 in MAM rats was significantly enhanced by the sub-chronic treatment with L-arginine (*p* < 0.05). The chronic treatment with NAC (250 mg/kg) was able to prevent the increase of locomotor activity caused by MK-801 in MAM rats. However, the NAC effect was partially blocked by L-arginine treatment in MAM rats (*p* < 0.05) (Figure 6).

#### 3.2.3 Immunohistochemistry for Detection of PV

**FIGURE 7:**
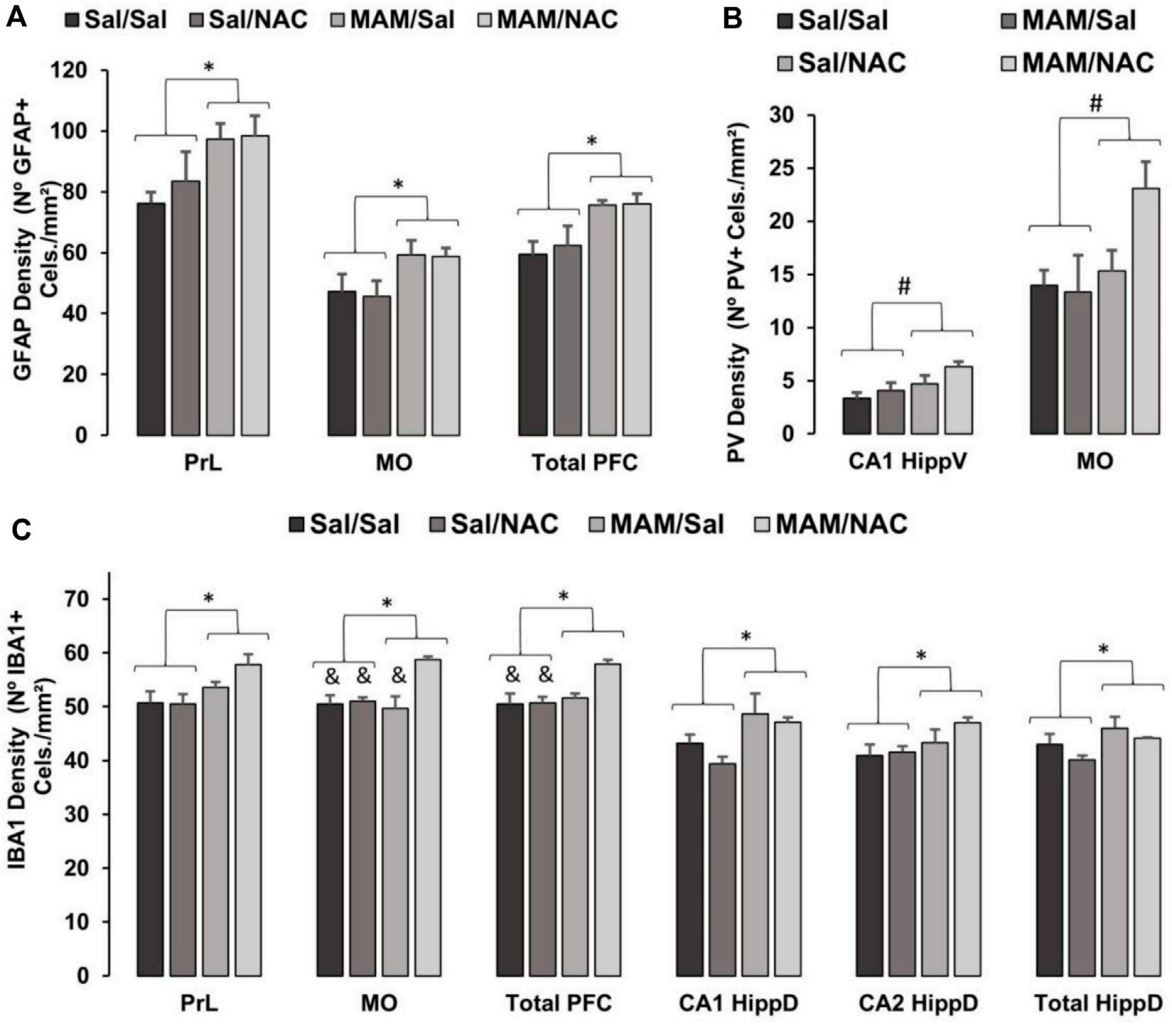
Effect of MAM and chronic treatment (15 days) with NAC (250 mg/kg) in GFAP positive astrocytes, PV positive interneurons and Iba1 positive microglia density (mean ± SE). DAB immunohistochemistry was performed to assess **(A)** GFAP, **(B)** PV, and **(C)** Iba1 in PFC and hippocampus of male offspring of MAM- or saline (SAL)-treated animals, subjected to chronic treatment with NAC or saline (Sal). The cell density was given by the sum of all cells divided by the total area in each region of interest, both data obtained by manual analysis using ImageJ software. **(A)** * indicates a significant difference compared to the SAL group in the prelimbic cortex (PrL), medial orbital cortex (MO), and total prefrontal cortex (PFC). **(B)** # indicates a significant difference for the NAC-treated group in MO and total PFC. **(C)** * indicates a significant difference compared to the SAL group in PrL, MO, total PFC, dorsal hippocampus (HippD), and its subregions CA1 and CA2. & indicates a significant difference compared to the MAM/NAC group (Duncan, *p* < 005).

**FIGURE 8:**
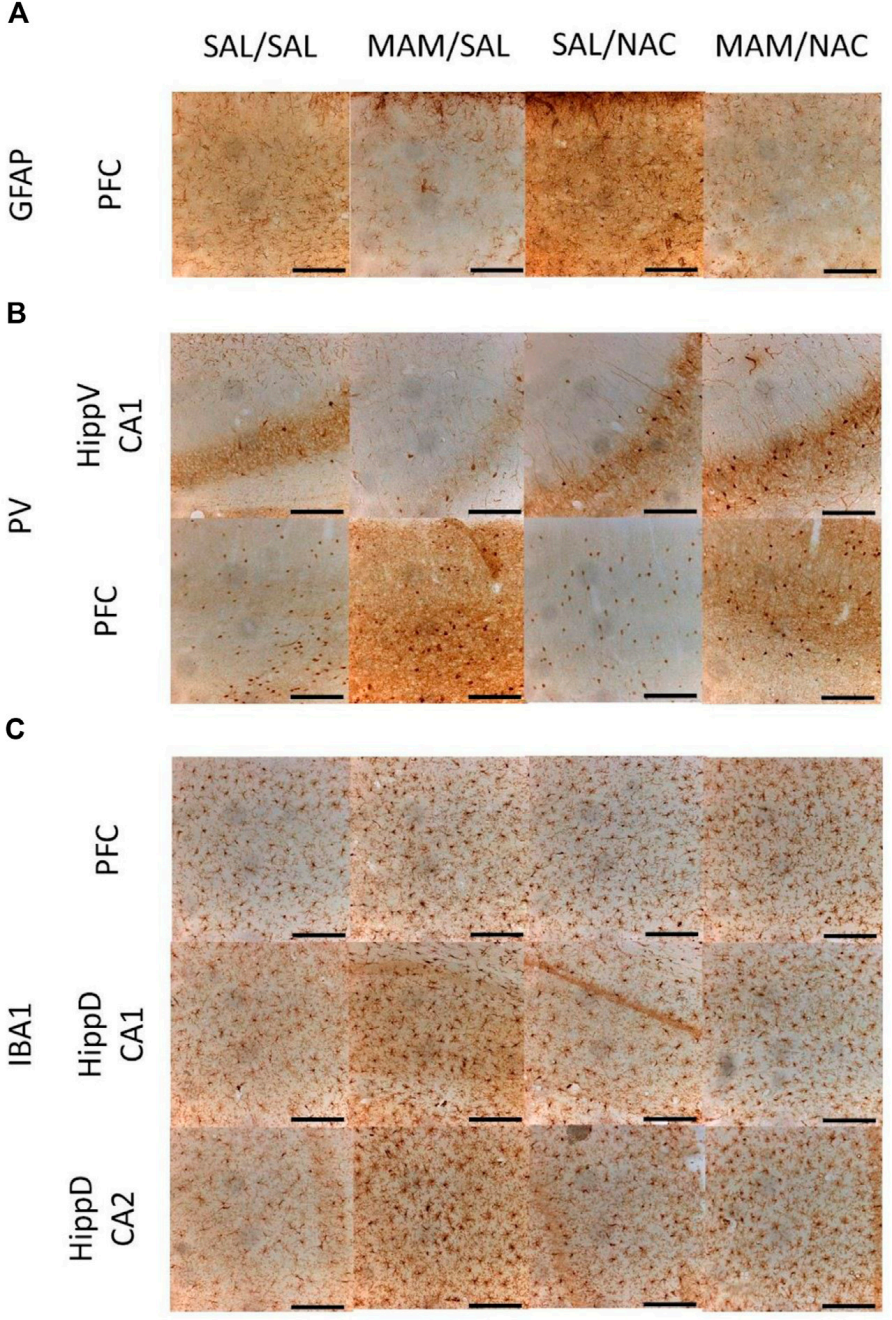
Immunohistochemistry for GFAP positive astrocytes, PV positive interneurons, and Iba1 positive microglia from the male offspring of MAM- or saline (SAL)- treated rats, under chronic treatment (15 days) with NAC (250 mg/kg) or saline (Sal). Representative photos obtained using an optical microscope with the 10× amplification of DAB immunostaining in 40 µm thickness slices show **(A)** increased expression of GFAP in the prefrontal cortex (PFC) of MAM rats, **(B)** increased expression of PV in PFC and CA1 from the ventral hippocampus (HippV) of rats treated with NAC, and **(C)** increased expression of Iba1 in PFC and in CA1 and CA2 of the dorsal hippocampus (HippD) in MAM rats (Duncan, *p* < 0.05). Scale bars represent 200 µm.

The two-way ANOVA test revealed no effect for treatment 1 on the PV positive interneuron density in any subregion of the PFC, dorsal, and ventral hippocampus (Figures 7, 8; Supplementary Table 1). However, a main effect for treatment 2 was observed in MO [F (1, 15) = 5.92; *p* < 0.05] and CA1 [F (1, 15) = 7.037; *p* < 0.05] of the ventral hippocampus and a marginally significant difference in the ventral subiculum [F (1, 15) = 4.117, *p* = 0.063], indicating an increase in the PV positive interneuron density in rats treated with NAC compared to controls, but no effect in any subregion of the dorsal hippocampus.

#### 3.2.4 Immunohistochemistry for Detection of GFAP

A two-way ANOVA test showed the main effect for treatment 1 on PrL [F (1, 15) = 7.1; *p* < 0.05], on MO [F (1, 15) = 5.773; *p* < 0.05] and total PFC [F (1, 15) = 10.14; *p* < 0.05], with an increase in the GFAP positive cell density in MAM-treated groups, compared to controls (Figures 7, 8; Supplementary Table 2). However, there was no effect of treatment 1 on dorsal CA1 (*p* = 0.143) and CA2 (*p* = 0.096), and ventral hippocampus [CA1 (*p* = 0.737), CA3 (*p* = 0.092), DG (*p* = 0.627), and total (*p* = 0.503)], only marginal effects in dorsal CA3 (*p* = 0.073) and DG (*p* = 0.068). There was no effect of treatment 2 in any subregion of PFC [PrL (*p* = 0.549), MO (*p* = 0.838), and total PFC (*p* = 0.726)], dorsal hippocampus [CA1 (*p* = 0.140), CA2 (*p* = 0.244), CA3 (*p* = 0.350), and DG (*p* = 0.154)], and ventral hippocampus [CA1 (*p* = 0.503), CA3 (*p* = 0.535), DG (*p* = 0.323), and total (*p* = 0.746)].

#### 3.2.5 Immunohistochemistry for Detection of Iba1

A two-way ANOVA test indicated the main effect of treatment 1 on PrL [F (1,15) = 6.814; *p* < 0.05], MO [F (1, 15) = 5.267; *p* < 0.05], total PFC [F (1, 15) = 4.795; *p* < 0.05], on the total dorsal hippocampus [F (1, 15) = 5.036; *p* < 0.05], dorsal CA1 [F (1, 15) = 10.315; *p* < 0.05], and dorsal CA2 [F (1, 15) = 4.523; *p* = 0.05]. In these regions, there was an increase in the density of Iba1 positive cells of MAM rats, compared to control rats (Figures 7, 8; Supplementary Table 3). This effect was not observed in dorsal CA3 (*p* = 0.585) or in any subregion of the ventral hippocampus [CA1 (*p* = 0.802), CA3 (*p* = 0.941), DG (*p* = 0.488), and total (*p* = 0.384)].

The analysis also showed the main effect of treatment 2 on MO [F (1, 15) = 10.563; *p* < 0.05] and total PFC [F (1, 15) = 4.795; *p* < 0.05], with an increase in Iba1 positive microglia density in NAC-treated groups (Figures 7, 8; Supplementary Table 3). However, no difference was found in the PrL [F (1, 15) = 1.074; *p* = 0.317], in any subregion of the dorsal hippocampus [CA1 (*p* = 0.216), CA2 (*p* = 0.260), CA3 (*p* = 0.128), DG (*p* = 0.257), and total (*p* = 0.149)] or ventral hippocampus [CA1 (*p* = 0.139), CA3 (*p* = 0.095), DG (*p* = 0.178), and total (*p* = 0.152)].

There was also an interaction between treatment 1 × treatment 2 on MO [F (1, 15) = 8, 14; *p* < 0.05]. The Duncan *post hoc* test revealed a higher Iba1 positive cell density in MAM/NAC compared to Sal/Sal in MO (*p* < 0.05) and PFC (*p* < 0.05), compared to Sal/NAC in MO (*p* < 0.05) and PFC (*p* < 0.05) and compared to MAM/Sal in MO (*p* < 0.05).

## 4 Discussion

To the best of our knowledge, the present study showed for the first time the effectiveness of the treatment with NAC during adulthood in restoring behavioral deficits in MAM rats. Both acute and chronic treatments with NAC were able to restore the deficit in the SI test, reduce both spontaneous and MK-801-induced hyperlocomotion, and reduce the increase in the ASR of most of the stimuli on the PPI test, in male offspring of rats treated with MAM. Additionally, sub-chronic treatment with the precursor of NO, L-arginine, prevented the NAC effect in recovering SI behaviors and in reducing the hyperlocomotion in these rats. Moreover, L-arginine potentiated hyperlocomotion caused by MK-801 in MAM rats, suggesting that NO mediates these behavioral changes, and the observed effects of NAC may involve its ability to act as an NO scavenger.

The deficits observed in MAM rats on the SI test are in accordance with previous findings with the MAM model (Flagstad et al., 2004; Le Pen et al., 2006; Hazane et al., 2009) and other animal models, such as the neonatal lesion in the ventral hippocampus (Genis-Mendoza et al., 2014) and the administration of the ammonitic epidermal growth factor (EGF) cytokine (Sotoyama et al., 2021). Our findings support the potential of the MAM animal model in mimicking negative symptoms observed in patients with schizophrenia (Wierońska et al., 2015; Potasiewicz et al., 2020).

The increased spontaneous (Le Pen et al., 2006; Ratajczak et al., 2015) or MK-801-induced hyperlocomotion tests (Le Pen et al., 2006; Pen et al., 2011) in our results also corroborate previous data using the MAM model (Lodge and Grace, 2012), and other animal models of schizophrenia, such as the neonatal lesion of the ventral hippocampus (Genis-Mendoza et al., 2014) and the spontaneously hypertensive rats (Almeida et al., 2014). Given that MK-801 causes the hypoactivation of NMDA receptors preferably in GABAergic interneurons (Thomases et al., 2013), it is conceivable that the spontaneous hyperlocomotion observed in MAM rats is due to both dopaminergic hyperactivity and glutamatergic hyperactivity (Hradetzky et al., 2012; Gomes et al., 2015a).

In contrast to some studies (Le Pen et al., 2006; Moore et al., 2006), we did not detect %PPI deficits in MAM rats. The lack of significant deficits in %PPI in MAM rats is related to a MAM effect of increasing all stimuli’s ASR, both to pulse and to prepulse + pulse. It is important to consider differently from these prior studies, which used white noise for the background, pulse, and for the three prepulses (Le Pen et al., 2006), we used pure tone for the prepulse stimuli and a higher prepulse intensity (Ewing and Grace, 2013; Wang et al., 2015). The use of higher intensity of prepulses, such as those used in the present study, did not demonstrate significant deficits in this test, but low intensity prepulses appear to be less discriminated by MAM rats, leading to deficits in PPI, as MAM rats appear to be able to discriminate between different sound intensities but fail to evoke gradual potential in response to different intensities of sound (Ewing and Grace, 2013).

Both acute and chronic treatments with NAC in adult rats were able to reduce hyperlocomotion caused by the NMDA antagonist and the deficit in SI in the MAM model. The treatment with NAC in juvenile animals or during adolescence had already been shown to be effective in other animal models of schizophrenia, such as the neonatal ventral hippocampus lesion (Cabungcal et al., 2014), the acute phencyclidine administration (Baker et al., 2008), knockout mice for GCLM (Otte et al., 2011), and the social isolation rearing model (Möller et al., 2013). Our data show that treatment with NAC in adulthood was also able to significantly improve behavioral deficits.

In the acute experiment, NAC at the dose of 250 mg/kg was able to revert the deficit in SI. For spontaneous locomotion, NAC, at doses of 250 mg/kg and 500 mg/kg, was able to revert the increased locomotor activity in the MAM group, and for hyperlocomotion induced by MK-801, all the three doses of NAC were able to revert the increased locomotion. The dose of 250 mg/kg was the most effective in reducing hyperlocomotion in MAM rats. The lower and higher doses (150 mg/kg and 500 mg/kg, respectively) of NAC were unable to reverse the deficit in SI and to reduce responsiveness to the NMDA antagonist. The lower dose of 150 mg/kg of NAC impaired these behaviors in the control groups. Thus, NAC treatment with the intermediate dose of 250 mg/kg was more effective in reversing deficits in the MAM model than with doses of 150 mg/kg and 500 mg/kg, showing a dose-dependent effect and a trend to an inverted “U” shaped dose-response curve.

The co-treatment with L-arginine (a precursor of NO) prevented the positive effect of NAC on SI and hyperlocomotion, indicating an involvement of the nitrergic system in the NAC mechanism of action. In previous studies, we showed that NAC could abolish the dopamine release and reuptake in the mesencephalic neuron culture induced by NO donors (Salum et al., 2008; Salum et al., 2016). Thus, it is possible that NAC prevents the increased dopaminergic activity in the ventral tegmental area and the dopaminergic induced hyperlocomotion in MAM rats (Hradetzky et al., 2012; Gomes et al., 2015b). Interestingly, the treatment with NG-nitro-L-arginine methyl, a NO synthase inhibitor, also prevented the hyperlocomotion and deficits in PPI caused by phencyclidine administration (Fejgin et al., 2008). Similarly, the treatment with NAC prevented the loss of PV positive interneurons and perineuronal net in the mice with the impaired GSH synthesis model of schizophrenia (Dwir et al., 2021). It is important to note that GSH can reduce the concentration of NO (Berk et al., 2013). We have observed that the acute treatment with the NO synthase inhibitor, L-NOARG, was able to significantly reduce MAM behavior deficits on %PPI and SI (under submission). The present results corroborate our previous data and reinforce that the NAC mechanism of action involves NO.

To further understand the mechanisms behind MAM and NAC treatments, immunohistochemistry was performed to investigate a possible involvement of neuroinflammation and oxidative stress in the MAM rats’ deficits. Our immunohistochemistry results for the animals subjected to chronic treatment with NAC, which did not receive L-arginine, showed indications of the ongoing inflammatory process. Those tests showed an increase in the density of GFAP cells in total PFC in MAM-treated groups, but no changes were observed in the dorsal and ventral hippocampus. No significant effects due to NAC were observed in the GFAP expression in any region. Furthermore, the Iba1 positive cell density was increased in all PFC subregions, total dorsal hippocampus, dorsal CA1, and CA2 of MAM rats. Unexpectedly, NAC increased the Iba1 cell density in MO and total PFC, in both SAL and MAM groups, compared to the rats treated with saline, but did not affect either dorsal or ventral hippocampus. The PV interneurons density was higher in MO and in CA1 of the ventral hippocampus of NAC-treated groups, but MAM treatment did not affect the PV expression in any region analyzed.

Dwir et al. (2020) showed that oxidative stress and neuroinflammation are connected by the activity of the redox-sensitive matrix metalloproteinase 9. In that study, they showed that the activity of metalloproteinase 9 enhanced the neuroinflammation in the GCLM knockout mice, connecting oxidative stress with neuroinflammatory processes. In the present study, there was a slight increase in Iba1 cell density of the hippocampus and PFC and in GFAP cell density of PFC from MAM rats. These alterations are indicatives of an ongoing inflammatory process in MAM rats. In the animal model of chronic treatment with MK-801 during 28 days beginning at PN42, an increase in the GFAP expression was observed, a decrease in resting microglia and an increase in activated microglia in medial PFC was also observed (Gomes et al., 2015b). However, another study with the same animal model showed a decrease in the GFAP marker on the PFC (Rahati et al., 2016). In the neonatal model of polyl:C, an increase in microglial activation was also observed in the PFC, hippocampus, and striatum from adolescence to adulthood (Ribeiro et al., 2013).

The present study did not consider the morphology of astrocytes and microglia, that is, the GFAP and Iba1 positive cells were not separated according to the characteristics of pro-inflammatory or anti-inflammatory processes. Considering that an increase in the expression of those protein markers is associated with both inflammatory responses (Howes and McCutcheon, 2017), it is not possible to determine whether the increases in microglia and astrocyte cell markers are related to pro- or anti-inflammatory mechanisms. Our results suggest that the effect of MAM can lead to a pro-inflammatory activation of microglia and astrocytes. On the other hand, the effect of NAC would lead to an anti-inflammatory activation in microglial cells. Further studies are necessary to uncover that question through the quantification of different inflammatory factors secreted by each cell in both inflammatory states.

The reduction of PV cells has previously been reported in the MAM model, specifically in the medial PFC and ventral subiculum of the hippocampus (Lodge and Grace, 2008) and this has been related to altered PFC and hippocampal during cognitive tasks, such as latent inhibition or PPI. Several studies with the MAM model presented contradictory results. Although some studies showed a decrease in PV expression in PFC (Lodge et al., 2009; Gastambide et al., 2012), in the ventral hippocampus (Lodge et al., 2009; Du and Grace, 2016) and dorsal hippocampus (Penschuck et al., 2006), others reported no changes in PV cells in the PFC (Penschuck et al., 2006) and dorsal hippocampus (Lodge et al., 2009). These different results could be related to the age of the animals when the cells were analyzed, as different results are found according to the age of the animal. In the MAM model, no difference was found in the PV cell density in PFC after 90 days of life (Penschuck et al., 2006), but a reduction was found in the 84 days studies in the medial PFC and ventral subiculum of the hippocampus of MAM-treated rats (Lodge et al., 2009). Although the age appears to be important, the number of cells expressing PV may not be correlated to the level of the PV protein and mRNA, as presented in a recent study where MAM failed to change the number of cells, but decreased levels of the PV protein in adulthood (Maćkowiak et al., 2019). The present samples were collected from animals at 90–91 days of life and did not show reduction in the PV positive cell density in MAM rats.

It is possible that a delayed maturation of the PV positive cells or a reduction in the expression of PV could also alter the function of PFC and hippocampus. Indeed, some studies with the knockdown of PV in rats produced similar behavioral deficits to those observed in the MAM model (Boley et al., 2014; Perez et al., 2019a). Therefore, NAC could also prevent a delayed maturation of perineuronal net and PV positive interneurons. It is interesting to note that the treatment with NAC before weaning was able to revert the increased dopaminergic activity in the ventral tegmental area and recover the loss of PV interneurons and the perineuronal net in the thalamic reticular nucleus in MAM rats (Zhu et al., 2021). Since the oxidative stress prevents the maturations of the perineuronal net and, consequently, the PV cells, NAC could act by preventing and recovering that delayed maturation. In fact, a study using mice with impaired synthesis of GSH revealed the ability of NAC in preventing the delay in the maturation of PV interneurons (Cabungcal et al., 2013). Consistently with this hypothesis, the treatment with NAC increased the density of PV interneuron in both PFC and hippocampus in the present study. Here, NAC was administered in adult rats from 75 to 90 days of life, indicating that NAC could increase PV interneurons even in adulthood.

The lack of effect of NAC treatment for glial cells suggests that its mechanism of action may not occur in the neuroinflammation promoted by MAM, but by reducing the oxidative stress and stimulating the maturations of PV interneurons. We, therefore, hypothesize that, in order to reverse the effects on GFAP and Iba1 cells, NAC treatment should be administered during the juvenile phase of these animals.

## 5 Conclusion

vOur results show that the antioxidant NAC was effective in recovering the behavioral deficits observed in MAM rats. Immunohistochemistry results suggest an ongoing inflammatory process in MAM rats. Additionally, our data give support to a potential antipsychotic effect of the antioxidant NAC. Although recovering the behavioral deficits, there was no effect on the inflammatory markers detected in MAM rats, suggesting that this may only be achieved with an early age antioxidant treatment. The present study corroborates previous findings suggesting that oxidative stress may have an important contribution for the schizophrenia symptomatology and support oxidative stress as a potential target for treatment.

## Data Availability

The raw data supporting the conclusion of this article will be made available by the authors, without undue reservation.
