# Supplementary material for: The Antioxidant N-Acetyl-L-Cysteine Restores the Behavioral Deficits in a Neurodevelopmental Model of Schizophrenia Through a Mechanism That Involves Nitric Oxide
Source: Front Pharmacol. 2022 Jul 12;13:924955. doi: 10.3389/fphar.2022.924955 (PMC9315304; doi:10.3389/fphar.2022.924955)
Supplement: Supplementary file 1 [file DataSheet1.PDF]

# *Supplementary Material*

## 1 Supplementary Tables

Supplementary Table 1: Effects of MAM and chronic treatment (15 days) with NAC in the density of parvalbumin positive interneurons (PV+) in dorsal (dHip) and ventral (vHip) hippocampus and prefrontal cortex (PFC). After behavioral testing the brain tissue from rats treated chronically with NAC were collected and the density of PV+ was analyzed by DAB immunohistochemistry. All positive immunoreactive cells in each region were obtained with the ImageJ software. The subregions evaluated were CA1, CA2, CA3, dentate gyrus (DG) and ventral subiculum (Sub) for dorsal and ventral hippocampus and medial orbital cortex (MO) and prelimbic cortex (PrL) from PFC. The total density was calculated by the sum of all cells divided by the total area of the region. *Indicates a significant difference between the SAL-treated group to NAC-treated group ($p < 0.05$). Data expressed as mean ± standard error.

| PV | | SAL/SAL | SAL/NAC | MAM/SAL | MAM/NAC |
|---|---|---|---|---|---|
| **dHip** | **CA1** | 7.24 (± 0.55) | 7.17 (± 0.99) | 7.4 (± 1.17) | 7.3 (± 0.41) |
| | **CA2** | 7.5 (± 0.06) | 7.12 (± 0.51) | 8.23 (± 0.13) | 7.99 (± 1.51) |
| | **CA3** | 6.03 (± 0.3) | 5.94 (± 0.67) | 6.73 (± 0.09) | 6.87 (± 0.54) |
| | **DG** | 2.64 (± 0.33) | 2.74 (± 0.5) | 1.81 (± 0.31) | 2.78 (± 0.66) |
| | **Total** | 5.23 (± 0.28) | 5.28 (± 0.66) | 4.81 (± 0.36) | 5.45 (± 0.47) |
| **vHip** | **CA1** | 3.32 (± 0.57)* | 4.68 (± 0.74) | 4.09 (± 0.84)* | 6.32 (± 0.48) |
| | **CA3** | 4.77 (± 0.77) | 5.30 (± 0.94) | 6.59 (± 0.73) | 6.50 (± 1.15) |
| | **DG** | 3.53 (± 0.68) | 3.25 (± 0.91) | 2.48 (± 1.11) | 6.79 (± 2.59) |
| | **Sub** | 2.01 (± 0.93) | 4.03 (± 1.13) | 2.23 (± 1.32) | 6.48 (± 3.11) |
| | **Total** | 3.57 (± 0.69) | 4.73 (± 0.80) | 4.83 (± 0.59) | 6.31 (± 0.51) |
| **PFC** | **MO** | 14.13 (± 2.23)* | 12.11 (± 1.09) | 9.96 (± 2.73)* | 16.21 (± 2.38) |
| | **PrL** | 13.94 (± 1.43) | 15.31 (± 1.96) | 13.34 (± 3.48) | 23.08 (± 2.54) |
| | **Total** | 14.01 (± 1.7) | 13.87 (± 1.44) | 11.58 (± 3.08) | 19.92 (± 2.45) |

Supplementary Table 2: Effects of MAM and chronic treatment (15 days) with NAC in the density of glial fibrillary acid protein positive astrocytes (GFAP+) in dorsal (dHip) and ventral (vHip) hippocampus and prefrontal cortex (PFC). After behavioral testing the brain tissue from rats treated chronically with NAC were collected and the density of PV+ was analyzed by DAB immunohistochemistry. All positive immunoreactive cell in each region was obtained with the ImageJ software. The subregions evaluated were CA1, CA2, CA3, dentate gyrus (DG) and ventral subiculum (Sub) for dorsal and ventral hippocampus and medial orbital cortex (MO) and prelimbic cortex (PrL) from PFC. The total density was calculated by the sum of all cells divided by the total area of the region. *Indicates a significant difference between the SAL-treated group to MAM-treated group ($p < 0.05$). Data expressed as mean ± standard error.

| **GFAP** | | **SAL/SAL** | **SAL/NAC** | **MAM/SAL** | **MAM/NAC** |
|---|---|---|---|---|---|
| **dHip** | **CA1** | 133.46 (± 6.72) | 105.22 (± 1.95) | 156.19 (± 29.79) | 133.31 (± 23.43) |
| | **CA2** | 160.67 (± 5.65) | 139.35 (± 8.19) | 195.11 (± 31.28) | 171.17 (± 28.95) |
| | **CA3** | 138.26 (± 10.75) | 120.4 (± 6.35) | 173.39 (± 32.76) | 155.95 (± 22.46) |
| | **DG** | 103.28 (± 8.36) | 83.38 (± 4.04) | 128.55 (± 24.13) | 109.34 (± 13.87) |
| | **Total** | 123.35 (± 7.23) | 100.87 (± 3.74) | 146.71 (± 27.45) | 127.93 (± 17.05) |
| **vHip** | **CA1** | 133.00 (± 6.37) | 143.58 (± 4.95) | 141.29 (± 8.8) | 139.84 (± 5.96) |
| | **CA3** | 113.26 (± 4.13) | 118.95 (± 4.98) | 136.29 (± 12.05) | 121.54 (± 7.97) |
| | **DG** | 128.26 (± 8.42) | 153.68 (± 12.09) | 146.81 (± 18.42) | 148.08 (± 14.95) |
| | **Sub** | 117.77 (± 7.99) | 120.56 (± 6.17) | 117.37 (± 7.41) | 114.62 (± 12.18) |
| | **Total** | 118.19 (± 4.92) | 130.20 (± 4.96) | 125.20 (± 8.23) | 120.56 (± 4.45) |
| **PFC** | **MO** | 76.23 (± 3.71)* | 83.47 (± 9.71)* | 97.32 (± 5.11) | 98.34 (± 6.67) |
| | **PrL** | 47.21 (± 5.72)* | 45.58 (± 5.29)* | 59.3 (± 4.82) | 58.74 (± 2.78) |
| | **Total** | 59.43 (± 4.23)* | 62.35 (± 6.43)* | 75.62 (± 1.64) | 76.05 (± 3.28) |

Supplementary Table 3: Effects of MAM and chronic treatment (15 days) with NAC in the density of binding adapter molecule 1 positive microglia (Iba1+) in dorsal (dHip) and ventral (vHip) hippocampus and prefrontal cortex (PFC). After behavioral testing the brain tissue from rats treated chronically with NAC were collected and the density of PV+ was analyzed by DAB immunohistochemistry. All positive immunoreactive cell in each region was obtained with the ImageJ software. The subregions evaluated were CA1, CA2, CA3, dentate gyrus (DG) and ventral subiculum (Sub) for dorsal and ventral hippocampus and medial orbital cortex (MO) and prelimbic cortex (PrL) from PFC. *Indicates a significant difference between the SAL-treated group to MAM-treated group ($p < 0.05$). The total

density was calculated by the sum of all cells divided by the total area of the region. Data expressed as mean ± standard error.

| Iba1 | | SAL/SAL | SAL/NAC | MAM/SAL | MAM/NAC |
|---|---|---|---|---|---|
| **dHip** | **CA1** | 43.17 (± 1.66)* | 39.44 (± 1.32)* | 48.71 (± 3.71) | 47.11 (± 0.92) |
| | **CA2** | 40.97 (± 2.02)* | 41.6 (± 1.11)* | 43.34 (± 2.38) | 46.97 (± 1.1) |
| | **CA3** | 42.81 (± 2.73) | 39.41 (± 0.89) | 44 (± 2.36) | 40.58 (± 0.53) |
| | **DG** | 43.41 (± 2.25) | 41.12 (± 0.53) | 46.86 (± 2.38) | 44.83 (± 0.82) |
| | **Total** | 43.04 (± 1.85)* | 40.1 (± 0.80)* | 46.02 (± 2.15) | 44.19 (± 0.18) |
| **vHip** | **CA1** | 46.62 (± 2.7) | 45.69 (± 1.39) | 49.88 (± 2.77) | 42.16 (± 4.07) |
| | **CA3** | 45.61 (± 1.65) | 43.39 (± 1.16) | 47.71 (± 0.7) | 43.43 (± 1.66) |
| | **DG** | 52.26 (± 1.97) | 51.2 (± 1.14) | 57.47 (± 2.13) | 50.18 (± 5.83) |
| | **Sub** | 51.31 (± 2.87) | 50.96 (± 0.70) | 45.57 (± 7.36) | 49.19 (± 6.94) |
| | **Total** | 48.30 (± 1.61) | 46.70 (± 0.55) | 48.72 (± 2.05) | 42.21 (± 5.54) |
| **PFC** | **MO** | 50.71 (± 2.15)* | 50.52 (± 1.87)* | 53.59 (± 1.03) | 57.83 (± 1.88) |
| | **PrL** | 50.47 (± 1.64)* | 51.07 (± 0.62)* | 49.64 (± 2.28) | 58.74 (± 0.55) |
| | **Total** | 50.51 (± 1.93)* | 50.71 (± 1.13)* | 51.62 (± 0.78) | 57.93 (± 0.82) |
